# Mechanisms of Class II correction induced by the crown Herbst appliance as a single-phase Class II therapy: 1 year follow-up

**DOI:** 10.1186/2196-1042-14-27

**Published:** 2013-09-11

**Authors:** Gundega Jakobsone, Dalia Latkauskiene, James A McNamara Jr

**Affiliations:** Department of Orthodontics, Lithuanian Unversity of Health Sciences, J.Luksos-Daumanto street 6, Kaunas, 50106 Lithuania; Department of Orthodontics, Riga Stradins University, Dzirciema street 20, Riga, LV 1007 Latvia; Department of Orthodontics and Pediatric Dentistry, Center for Human Growth and Development, University of Michigan, 1011 N.University Avenue, Ann Arbor, MI 48109-1078 USA

**Keywords:** Herbst appliance, Cephalometrics, Class II malocclusion, Functional jaw orthopedics

## Abstract

**Background:**

The objective of this study is to evaluate the skeletal and dentoalveolar effects of the crown Herbst appliance used alone for a single phase of therapy followed by a 1-year observation period.

**Methods:**

Forty patients (mean age 13.6 ± 1.3 years) with a stable Class I occlusion 1 year following the treatment with the crown Herbst appliance were selected from a prospective sample of 180 consecutively treated Class II patients. No other appliances were used during treatment or during the follow-up period. The dentoskeletal changes were compared with a matched sample of untreated Class II subjects (mean age 13.9 ± 1.6 years). Lateral cephalograms were taken before treatment, after Herbst treatment (1 year), and after 1-year follow-up. Overcorrection was avoided intentionally.

**Results:**

Treatment produced an increase in mandibular length, a decrease in ANB angle, and a restriction in the vertical growth of posterior maxilla. The maxillary molars moved backward and tipped distally. The lower incisors proclined markedly, and the upper incisors became retroclined. During the follow-up period, the changes primarily were dentoalveolar in nature, with marked rebound of the upper molars and lower incisors, resulting in some increases in overbite and overjet.

**Conclusions:**

The occlusal correction of Class II malocclusion observed 1 year after the crown Herbst appliance as a single-phase therapy was achieved primary due to the dentoalveolar changes and only limited skeletal change occurred.

## Background

Class II malocclusion is one of the most common orthodontic problems, as it occurs in about one third of the United States population [1]. A wide range of fixed and removable functional appliances is available for correction of Class II skeletal and occlusal disharmonies; however, their modes of action are diverse.

The banded-type Herbst appliance was reintroduced by Pancherz [2] as a Class II treatment modality. During the last three and a half decades, the Herbst appliance has become increasingly popular, mostly due to good patient compliance [3]. A number of studies have reported very high success rates in Class II correction by the Herbst appliance [4–6], usually followed by fixed appliance therapy [5, 6].

High manufacturing expenses [3] and high band breakage rate [7] have been reported as disadvantages of the Herbst appliance. To overcome these drawbacks, in 1980, Langford introduced the stainless steel crown Herbst (cHerbst) modification [8]. Although several controlled studies have shown an increase in mandibular length as a result of treatment with different modifications of the Herbst appliance [4–6, 9–11], the treatment effects produced by each appliance should be examined separately because of the different modes of action due to the specific construction and application.

A systematic review by Barnett et al. [12] has shown that data on the treatment effects of the crown or banded type of the Herbst appliance is limited. A recent prospective study by Latkauskiene et al. [13] on a sample of 180 consecutive patients showed very good compliance and 100% Class II malocclusion correction with the cHerbst. These patients did not receive any type of retention appliance and were followed for additional 1 year posttreatment. The occlusion of 77% of the patients remained stable.

The present report is the natural extension of the clinical study described above [13]. This follow-up study was designed to evaluate the skeletal and dentoalveolar effects of the crown Herbst appliance used as a single appliance for a single phase therapy. The study had the following objectives:

- to analyze the skeletal and dentoalveolar components of Class II correction during active treatment.
- to determine the skeletal and dental changes during a period when no retention procedures were used.
- to compare the treatment and posttreatment changes against those of the untreated Class II individuals matched to the treated patients on the basis of the level of skeletal maturation.

## Methods

The sample considered in the present study was derived from a prospective sample of 180 patients described earlier [13]. All patients were treated by the same operator (D.L.) in one private orthodontic practice with a standardized bite-jumping appliance, the stainless steel crown Herbst (cHerbst; Ormco Corporation, 1717 West Collins Avenue, Orange, CA) as the only appliance. The management of the appliance during treatment and follow-up was described in detail in a previous publication [13].

In all patients, the appliance was left in place for 12 months, and a Class I occlusion was achieved in all cases. One year after the treatment, 72 patients had stable Class I occlusion achieved with cHerbst as the only appliance. From the initial sample of 180 patients, 5 did not finish the active phase of treatment, 58 chose to start fixed appliance therapy after the first phase of treatment, 24 did not show up for the check-up visits, and 21 patients had relapse either 6 months (19 patients) or 12 months (2 patients) after treatment. Seven patients refused to have the cephalogram taken at the end of the follow-up. Thus, lateral cephalograms of 65 patients with stable Class I occlusions were available at the following observation periods: before treatment (T1), after active phase of treatment (T2), and at 1-year follow-up (T3).

It should be noted that the cHerbst appliance was managed slightly different in this study than in the typical contemporary clinical setting in which fixed appliance therapy follows functional jaw orthopedic treatment immediately. These patients were treated in a private practice setting, and it was intended that this group of patients was going to be treated without a second phase of fixed appliance treatment; every attempt was made to allow the occlusion to settle properly during and after the time that the stainless steel crown Herbst appliance was worn. The bite registration was taken in an incisal edge-to-edge incisor relationship. The cusps of the upper first premolars were positioned between the lower first and second premolars to help facilitate the natural vertical settling of the occlusion. The appliance was removed after 12 months, according to the standardized treatment protocol.

The cervical vertebral maturation (CVM) method [14] was used to evaluate the skeletal maturity of the patients. CVM stages on pretreatment and posttreatment lateral cephalograms were assessed by Professor Lorenzo Franchi (University of Florence). Forty patients (20 male, 20 female) with the mean age 13.6 ± 1.3 were judged as growing subjects and included in the study group. The patients started treatment either at puberty (CS3, *n* = 26) or at postpubertal stages (CS4 or CS5, *n* = 14), and they finished treatment either at a postpubertal stage (CS5, *n* = 26) or at the end of active growth (CS6, *n* = 14).

The control group consisted of 18 subjects (11 males, 7 females) who were selected from the longitudinal records of the University of Michigan Elementary and Secondary School Growth Study and the Denver Child Growth Study. The mean age of the control group was 13.8 ± 1.6 years. The control group was matched to the treated group as to the skeletal maturity at all observation periods (Table 1).

**Table 1:** **Demographics for the treated and untreated Class II groups**

				Age at T1, years	Age at T2, years	Age at T3, years
	Number	Female	Male	Mean (SD)	Mean (SD)	Mean (SD)
cHerbst group	40	20	20	13.6 (1.3)	14.8 (1.4)	15.9 (1.4)
Control group	18	7	11	13.9 (1.6)	14.7 (1.4)	15.7 (1.4)

### Cephalometric analysis

Cephalograms were taken with the Frankfort horizontal parallel to the floor with teeth in occlusion and lips relaxed. All cephalograms were hand-traced by the same examiner (G.J.) on acetate paper. The tracings were digitized with Dentofacial Planner Plus software (Dentofacial Software, Toronto, Canada) on a computer with a digitizing screen (Numonics Cooperation, Montgomeryville, USA). All cephalograms were adjusted to 0% enlargement.

Two cephalometric landmarks, frontomaxillary nasal suture (FMN) and T-point (the most superior point of the anterior wall of sella turcica, at the junction with tuberculum sellae) have been used by Franchi et al. [5] as the reference points for studies of growing individuals. Skeletal and dentoalveolar changes were recorded using a grid system: the *x*-axis was drawn through T and FMN points, while the *y*-axis was constructed perpendicular to *x*-axis through T-point.

The following landmarks were digitized, and the changes were assessed in the constructed grid system: condylion (Co), gonion (Go), point A, point B, pogonion (Pg), menton (Me), upper first molar mesiobuccal cusp tip (ms), lower first molar mesiobuccal cusp tip (mi), upper incisor tip (is), and lower incisor tip (ii).

The following linear and angular measurements were performed:

- Linear measurements: overjet, overbite, mandibular length (Co-Gn), midfacial length (Co-A), ramus length (Co-Go), and mandibular body length (Go-Me).
- Angular measurements: sella-Nasion-point A (SNA), sella-Nasion-point B (SNB), point A-Nasion-point B (ANB), SNPog, Co-Go-Me, upper incisor inclination to T-FMN line, lower incisor inclination to mandibular plane (ml), upper molar axial line (mesiobuccal cusp to mesiobuccal root) inclination to T-FMN line (upper molar tipping), maxillary plane to T-FMN line, and mandibular plane to T-FMN line.

Class II correction was calculated as described earlier by Pancherz [4], but the *y*-axis served as the vertical reference line.

### Statistical analysis

Statistical analysis was performed with SPSS for Windows software (SPSS, Chicago, IL, USA). The data were tested for normality of distribution (Shapiro-Wilk test) and equality of variances (Levene's test). Independent *t* tests were used to detect the differences in the skeletal and dentoalveolar characteristics between the treated and control groups at the start of treatment as well as in the changes of the measurements during the observation intervals (T1-T2, T2-T3, and T1-T3).

The power of the study was calculated considering the ANB angle as a sensitive variable for the assessment of the orthopedic effects of Class II treatment. With a clinically significant change of 2.0 degrees in the ANB angle, a standard deviation (SD) for this angle of 1.3° (as derived from a previous study on the effects of the stainless steel crown Herbst) [15] and alpha of 0.05, the calculated power for the independent sample *t* test exceeded 0.90.

### Method error

Twenty cephalograms selected randomly from the treated sample were retraced and digitized by the same investigator (G.J.) on two separate occasions at least 2 weeks apart to calculate the method error with Dahlberg's formula [16] and to assess the intraclass correlation coefficient (ICC). The method error for linear measurement ranged from 0.3 mm (lower incisor to T-FMN line) to 0.7 mm (lower mandibular cusp to T-FMN line) and for angular measurements, −0.2 degrees for ANB angle to 1.2 degrees for lower incisor inclination. ICC for linear measurements varied from 0.949 for overjet to 0.998 for point B to the *y*-axis. For angular measurements, the values ranged from 0.962 (for maxillary plane to T-FMN line) to 0.988 (for SNA and SNB angles).

Written informed consent was obtained from the patients for the publication of this report. The approval of conducting the present research was received from Kaunas Regional Committee of Ethics for Biomedical Research (Kauno Regioninis Biomedicininu Tyrimu Etikas Komitetas), Nr. BE-2-34.

## Results

Pretreatment characteristics of the groups are summarized in Table 2. No significant differences were found at T1 between the treated and control samples with the exception of midfacial length (Co-A) and of the inclination of the upper incisors to T-FMN (Is to T-FMN) that were significantly greater in the treatment group. There was no statistically significant difference in age between the groups at any of the time points. The dental and skeletal changes during the observation intervals are presented in Tables 3, 4, and 5.

**Table 2:** **Pretreatment characteristics of the study subjects compared with matched untreated Class II individuals**

Variable	Study group	Control group	***P*** value
	***n*** = 40	***n*** = 18	
Overjet (mm)	5.5 (2.2)	5.5 (2.4)	0.130
Overbite (mm)	5.6 (1.3)	4.5 (2.0)	0.758
SNA (degrees)	81.5 (2.9)	81.1 (3.8)	0.605
SNB (degrees)	76.7 (2.4)	76.6 (3.2)	0.910
SNPog (degrees)	78.1(2.6)	78.0 (3.3)	0.886
ANB (degrees)	4.8 (1.9)	4.5 (1.5)	0.449
Co-A (mm)	86.6 (4.7)	83.6 (6.6)	0.045
Co-Gn (mm)	108.1 (5.4)	105.8 (7.3)	0.187
is to T-FMN (degrees)	111.5 (8.2)	104.4 (8.2)	0.003
ii to ml (degrees)	99.8 (7.0)	98.7 (7.6)	0.599
Co-Go-Me (degrees)	121.5 (4.9)	119.8 (4.1)	0.178

Mean values and standard deviations (SD) in parenthesis.

**Table 3:** **Descriptive statistics and statistical comparisons of the T1-T2 changes**

Cephalometric measures	cHerbst group		Control group		Net change	***P*** value
	Mean	SD	Mean	SD		
Skeletal measures						
Co hor (mm)	−0.9	1.2	−0.4	1.3	−0.5	0.198
Co ver (mm)	0.3	1.2	0.9	1.3	−0.6	0.098
A hor (mm)	0.5	1.0	0.9	1.2	−0.4	0.161
B hor (mm)	1.7	1.9	1.0	1.7	0.7	0.185
Pg hor (mm)	1.9	2.1	1.4	2.1	0.5	0.383
Me ver (mm)	3.4	1.6	2.6	2.7	0.8	0.193
Co-A (mm)	1.6	1.5	1.3	1.8	0.3	0.593
Co-Gn (mm)	4.0	1.8	2.7	2.4	1.3	0.026
Co-Go (mm)	2.7	3.7	1.1	1.5	1.6	0.071
Go-Me (mm)	1.4	3.1	1.3	2.7	0.1	0.913
SNA (°)	−0.3	0.9	0.2	1.1	−0.5	0.142
SNB (°)	0.8	1.0	0.1	0.8	0.7	0.014
SNPog (°)	0.7	0.9	0.3	0.6	0.4	0.122
ANB (°)	−1.1	1.0	0.0	0.7	−1.1	0.000
pp to T-FMN (°)	0.7	1.2	−0.5	1.9	1.2	0.005
ml to T-FMN (°)	0.3	1.9	0.1	2.0	0.2	0.684
Co-Go-Me (°)	0.6	2.8	0.7	1.7	−0.1	0.890
Dental measures						
Overjet (mm)	−2.7	1.9	0.0	0.8	−2.7	0.000
Overbite (mm)	−2.7	1.3	−0.4	0.7	−2.3	0.000
ms hor (mm)	−1.6	1.6	1.1	1.7	−2.7	0.000
ms ver (mm)	1.4	1.2	1.7	1.9	−0.3	0.501
Upper molar tipping (°)	−5.7	4.5	1.4	4.1	−7.1	0.000
mi hor (mm)	3.6	1.9	1.1	1.7	2.5	0.000
mi ver (mm)	2.6	1.3	1.6	2.0	1.0	0.031
is hor (mm)	0.1	1.8	1.0	1.3	−0.9	0.076
is ver (mm)	1.6	1.4	1.1	1.6	0.5	0.291
ii hor (mm)	2.9	1.7	1.0	1.6	1.9	0.000
ii ver (mm)	4.3	1.7	1.5	2.0	2.8	0.000
Upper incisor inclination (°)	−2.3	4.6	0.3	2.1	−2.6	0.023
Lower incisor inclination (°)	4.6	4.1	−0.8	3.4	5.4	0.000

**Table 4:** **Descriptive statistics and statistical comparisons of the T2-T3 changes**

Cephalometric measures	cHerbst group		Control group		Net change	***P*** value
	Mean	SD	Mean	SD		
Skeletal measures						
Co hor (mm)	−0.6	1.1	−0.5	1.2	0.1	0.876
Co ver (mm)	0.3	1.1	0.0	1.3	0.3	0.489
A hor (mm)	0.8	1.5	0.3	1.8	0.5	0.276
B hor (mm)	0.7	1.8	0.9	1.8	−0.2	0.752
Pg hor (mm)	0.7	2.1	1.0	2.1	−0.3	0.598
Me ver (mm)	1.4	2.3	1.2	1.0	0.2	0.681
Co-A (mm)	1.5	1.7	1.0	1.3	0.5	0.228
Co-Gn (mm)	1.8	2.2	1.6	1.6	0.2	0.737
Co-Go (mm)	1.6	2.7	2.0	2.5	−0.4	0.556
Go-Me (mm)	1.0	2.7	0.8	2.0	0.2	0.868
SNA (°)	0.4	1.0	−0.3	1.0	0.7	0.042
SNB (°)	0.3	0.9	0.1	0.8	0.2	0.504
SNPog (°)	0.7	0.9	0.1	0.9	0.6	0.482
ANB (°)	0.1	0.9	−0.4	0.8	0.5	0.118
pp to T-FMN (°)	0.0	1.0	0.1	1.8	−0.1	0.880
ml to T-FMN (°)	−0.7	1.5	−0.8	1.7	0.1	0.740
Co-Go-Me (°)	−0.5	1.9	−0.6	1.6	0.1	0.875
Dental measures						
Overjet (mm)	0.7	1.0	−0.5	1.2	1.2	0.000
Overbite (mm)	1.4	1.2	0.0	0.7	1.4	0.000
ms hor (mm)	2.1	2.5	0.8	1.7	1.3	0.055
ms ver (mm)	1.5	1.6	0.9	1.0	0.6	0.077
Upper molar tipping (°)	4.4	4.5	1.7	3.6	2.7	0.028
mi hor (mm)	0.4	1.7	0.9	1.6	−0.5	0.342
mi ver (mm)	0.8	1.8	0.6	1.0	0.2	0.774
is hor (mm)	0.6	1.8	0.2	1.6	0.4	0.418
is ver (mm)	1.2	1.6	0.5	1.0	0.7	0.110
ii hor (mm)	−0.1	1.5	0.7	1.4	−0.8	0.075
ii ver (mm)	−0.2	1.9	0.5	0.8	−0.7	0.192
Upper incisor inclination (°)	−0.6	3.3	−1.0	2.6	0.4	0.666
Lower incisor inclination (°)	−3.2	3.9	−0.6	2.5	−2.6	0.013

**Table 5:** **Descriptive statistics and statistical comparisons of the T1-T3 changes**

Cephalometric measures	cHerbst group		Control group		Net change	***P*** value
	Mean	SD	Mean	SD		
Skeletal measures						
Co hor (mm)	−1.5	1.5	−1.0	1.5	−0.5	0.240
Co ver (mm)	0.6	1.5	0.9	1.7	−0.3	0.401
A hor (mm)	1.3	1.8	1.3	2.0	0.0	0.886
B hor (mm)	2.4	2.6	1.8	2.5	0.6	0.467
Pg hor (mm)	2.5	2.9	2.4	3.1	0.1	0.837
Me ver (mm)	4.9	3.0	3.8	3.4	1.1	0.221
Co-A (mm)	3.2	2.3	2.3	2.1	0.9	0.172
Co-Gn (mm)	5.8	2.9	4.3	3.4	1.5	0.082
Co-Go (mm)	4.4	3.8	3.1	2.5	1.3	0.214
Go-Me (mm)	2.4	3.4	2.1	3.0	0.3	0.752
SNA (°)	0.1	1.2	−0.1	1.6	0.2	0.549
SNB (°)	1.1	1.1	0.2	1.3	0.9	0.014
SNPog (°)	1.0	1.1	0.4	1.3	0.6	0.104
ANB (°)	−1.0	1.2	−0.4	0.7	−0.6	0.035
pp to T-FMN (°)	0.7	1.1	−0.4	1.8	1.1	0.005
ml to T-FMN (°)	−0.4	2.1	−0.7	1.7	0.3	0.488
Co-Go-Me (°)	0.0	2.7	0.1	2.5	−0.1	0.923
Dental measures						
Overjet (mm)	−2.0	1.6	−0.6	1.6	−1.4	0.003
Overbite (mm)	−1.4	1.0	−0.4	1.0	−1.0	0.000
ms hor (mm)	0.6	2.7	2.0	2.5	−1.4	0.065
ms ver (mm)	3.0	1.9	2.5	2.3	0.5	0.332
Upper molar tipping (°)	−1.2	4.8	3.1	5.1	−4.3	0.003
mi hor (mm)	4.1	2.3	2.0	2.4	2.1	0.003
mi ver (mm)	3.6	2.1	2.3	2.3	1.3	0.078
is hor (mm)	0.8	2.4	1.2	2.1	−0.4	0.527
is ver (mm)	2. 8	1.9	1.6	2.0	1.2	0.041
ii hor (mm)	2.8	2.1	1.7	2.1	1.1	0.058
ii ver (mm)	4.2	2.1	2.0	2.3	2.2	0.001
Upper incisor inclination (°)	−2.9	4.9	−0.6	2.9	−2.3	0.081
Lower incisor inclination (°)	1.4	4.6	−1.4	4.0	2.8	0.026

### Skeletal changes

The Herbst appliance had no effect on the sagittal position of the maxilla. No significant change was found in the horizontal and vertical positions of the maxillary and mandibular landmarks with respect to the stable basicranial reference system. During active treatment, the total mandibular length (Co-Gn) showed a statistically greater increase (1.3 mm, Table 3) with respect to the controls, an increase that was maintained in the posttreatment period (T1-T3 change, 1.5 mm), though not at a statistically significant level (Table 5). This alteration in the amount of mandibular growth during active treatment was associated with a significantly greater increase in the SNB angle (0.7°) and a significantly greater decrease in the ANB angle (−1.1°) that remained stable in the follow-up (T1-T3 change, 0.9°, and −0.6°, respectively).

As for the skeletal vertical parameters, the palatal plane angle (pp to T-FMN) showed a significant clockwise rotation of 1.2° in the treated vs. the control group; this effect was maintained throughout the posttreatment period. No significant change was recorded in the inclination of the mandibular plane to FMN or in the Co-Go-Me angle.

### Dental changes

The treatment group showed a significant reduction of overjet (−2.7 mm) and correction of overbite (−2.3 mm) with respect to the controls. Although during the posttreatment period, a slight relapse was recorded for both overjet and overbite, statistically significant corrections were still present when considering the T1-T3 period (−1.4 and −1.0 mm, respectively).

The upper first molars moved backward at a statistically significantly amount (2.7 mm) and tipped distally (7.1°) in comparison to the untreated group. Although a major portion of the tipping rebounded during the follow-up (to 2.7°), the Class I relationship of the buccal segments was preserved, and the upper molars remained significantly tipped back by 4.6° in the treated sample with respect to the controls. During treatment, the upper incisors showed a significant uprighting (−2.6°) that was maintained when considering the T1-T3 follow-up period (−2.3°).

During active treatment (T1-T2), the lower first molars moved forward significantly by 2.5 mm and extruded significantly by 1.0 mm with respect to the controls. When considering the overall observation period (T1-T3), the lower molars still showed a significant forward movement of 2.1 mm while the extrusion of the lower molars was not statistically significant in comparison to control values. The lower incisors proclined significantly (4.6°) in the treated sample as opposed to the slight uprighting (−0.8°) in the controls during T1-T2. During the T2-T3 period, the lower incisors uprighted (−3.2°) as did the controls (−0.6°); when considering the overall observation period (T1-T3), the proclination of the lower incisors was still significantly greater in the treated group with respect to the controls by 2.8°. Although the appliance exerted a significant vertical force to the upper molars, no significant change in their vertical position was recorded. Vertical changes of the lower incisors in the study sample could be attributed to their proclination.

### Class II correction mechanisms

**Figure 1:**
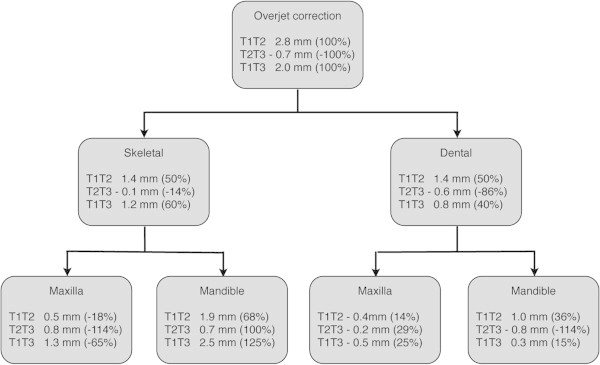
**Comparison of skeletal and dental changes contributing to overjet correction with the stainless steel crown Herbst appliance.**

**Figure 2:**
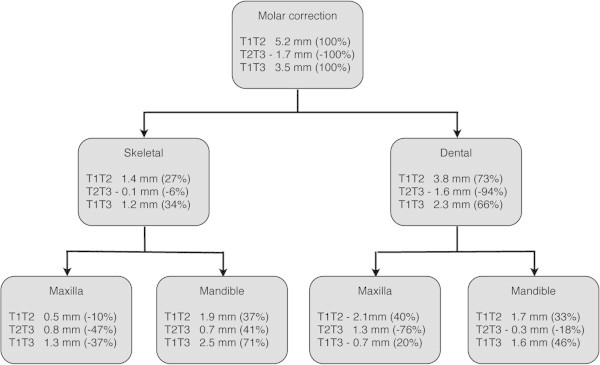
**Comparison of skeletal and dental changes contributing to sagittal molar correction with the stainless steel crown Herbst appliance.**

The components of overjet and molar relationship correction during treatment and posttreatment changes are summarized in Figures 1 and 2.

In the correction of molar relationship during treatment, an important parameter was the distal movement of the upper molars, which rebounded almost completely during the follow-up period. Thus, at the end of the observation period, the molar relationship was maintained by further forward growth of the mandible and forward movement of the lower molars.

The appliance produced an overjet reduction by changing the dental and skeletal parameters equally. During the follow-up period, the changes primarily were dentoalveolar in nature.

## Discussion

The aim of the present study was to evaluate Class II correction mechanisms with the cHerbst appliance by comparing the changes in the treated group with the growth changes of untreated Class II individuals. In the present study, only the subjects with stable Class I relationship 1 year after the single active treatment phase with cHerbst were included [13]. It also would have been of interest to analyze the unsuccessful cases. However, from the subjects who returned for follow-up visits, only eight growing patients experienced relapse, a number that was too small to evaluate statistically. Thus, this study evaluated the treatment effects of the cHerbst appliance alone because no additional orthodontic appliances (including retainers) were used during treatment stage as well as during the 12-month follow-up period.

A considerable advantage of the study was that the treatment of the consecutive subjects was conducted by a single operator who followed a strict protocol. The operator was not involved in the analysis of the data, thus reducing bias of the study. The treated and control groups were matched on the basis of skeletal maturity. Although historical control groups may present with limitations [17], in the current study, the use of historical controls was necessitated by the lack of ethical reasons to leave Class II patients untreated at the circumpubertal growth period, a stage of development that is known to represent the optimal time for orthopedic modifications [6].

### Skeletal changes

The increase in mandibular length induced by cHerbst therapy was found to be similar to that reported in some other studies [15, 18–20]. During the follow-up period, the mandibular length increased with the same amount in both the treatment and the control groups. Thus, at the end of the 1-year observation period, the net increase of the mandible in the study group was 1.5 mm more compared to the controls; however, this difference was not significant statistically. This finding is consonant with the study by Pancherz and Fackel [21] in which the band Herbst appliance was used as the only appliance. When comparing the skeletal and dentoalveolar changes 31 months before and after treatment, they concluded that the Herbst appliance had only a temporary impact on the existing craniofacial growth pattern. Nevertheless, the occlusal relationships were improved, and the Class I relationship was maintained 1 year after treatment.

In the majority of the studies in which the first phase treatment with the Herbst appliance was followed by fixed appliances, a statistically significant net increase in mandibular length at the end of treatment was recorded [5, 6, 10, 15]. In most of those studies, an acrylic Herbst was used along with stepwise activation of the appliance [5, 6, 10]. The design and construction of the acrylic Herbst could suggest better dentoalveolar anchorage as well as the inhibition of vertical development because of the interocclusal coverage of the splint. In a direct comparison of the acrylic splint and stainless steel crown Herbst appliance, however, the investigation of Burkhardt and co-workers [20] indicated that the two appliance designs produced similar changes in horizontal and vertical skeletal position.

The moderate effect on mandibular length also can be explained at least partially by the difference in treatment strategies. Because previous studies on the factors influencing relapse after Herbst treatment [22, 23] emphasized the importance of attaining a stable occlusion at the end of Herbst appliance therapy, the treatment objective of the present sample was to establish Class I relationship during the active phase of treatment rather than to ‘overtreat’ the occlusion in to a ‘super Class I’ relationship. At the start of treatment, the appliance was activated to an edge-to-edge relationship, and during treatment, the buccal segments were controlled for settling, as mentioned earlier. As the lower incisors moved forward and the upper incisors retroclined, no further activation was possible in order to avoid the creation of a negative overjet.

Several previous studies have suggested that the following treatment strategy should be employed to induce increase in condylar growth: (1) stepwise advancement, (2) a 6-month duration for each instance of advancement, and (3) initial advancement of at least 5 mm [24–26]. In the present sample, not all of these requirements could be realized due to the predominant dentoalveolar effects of appliance. Although the cHerbst appliance had no significant effect in restraining sagittal growth of the maxilla, a significant restriction in vertical growth of the posterior maxilla was observed, an observation that also been reported in other studies of the Herbst appliance [4, 5, 10, 11].

### Dentoalveolar changes

Similar to other studies [9, 22], significant dental relapse/rebound was recorded during the follow-up period, even though a Class I relationship was maintained. Interestingly, in overjet correction, skeletal changes contributed slightly more, while molar correction was achieved mainly by dental movements. Other studies have reported comparatively equal contribution of both components [4, 5, 11].

The Herbst appliance was shown to have a headgear effect, and the upper molars were distalized and tipped backward significantly. This finding was in agreement with other studies [4, 5, 10, 11, 18]. During the follow-up period, the upper molars tended to rebound to a more mesial position by 1.3 mm. However, a Class I relationship of the buccal segments was maintained. A similar observation was noted by Burkhardt et al. [20], who explained this phenomenon to be a result of a favorable growth pattern and dentoalveolar compensation.

The appliance produced a moderate lingual tipping of the upper incisors, while the lower incisors were proclined significantly. Similar findings also have been demonstrated in some other samples [4, 5, 10, 11]. Apparently, such factors as the method of Herbst appliance anchorage in the mandible [27] or the amount of initial activation [22] does not affect the proclination of the lower incisors.

One year after treatment, the lower incisors rebounded by 2.6°, resulting in overjet and overbite correction loss. Pancherz and Hansen [27] found that 80% of the lower incisors proclination recovered within 12 months; however, they noted that rebound of the incisors was not associated with significant crowding. An increase in overjet 3 years after successfully treated Class II malocclusion also was reported for the twin block appliance [28]. The appliance design facilitated forward movement of the molars within the mandible that was slightly greater than that reported in other samples [4, 5, 11]. Forward movement of the lower molars assisted in molar relationship correction because the overall mandibular forward movement was slightly smaller than that reported in previous studies [4, 5, 11].

## Conclusions

- One year after treatment with the stainless steel crown Herbst appliance, the correction of the molar relationships was achieved primary due to dentoalveolar changes (66% dental vs. 34% skeletal).
- Substantial distalization of the upper molars was achieved without significant effect on the upper incisors; therefore, the appliance could be recommended in dentoalveolar Class II cases, especially with crowding in the upper arch.
- When clinically significant mandibular advancement is desirable, other treatment protocols or orthognathic surgery should be considered.
